# Leveraging human genomic information to identify nonhuman primate sequences for expression array development

**DOI:** 10.1186/1471-2164-6-160

**Published:** 2005-11-15

**Authors:** Eliot R Spindel, Mark A Pauley, Yibing Jia, Courtney Gravett, Shaun L Thompson, Nicholas F Boyle, Sergio R Ojeda, Robert B Norgren

**Affiliations:** 1 Division of Neuroscience, Oregon National Primate Research Center, Beaverton, OR 97006, USA; 2 College of Information Science & Technology, University of Nebraska at Omaha, Omaha, NE, 68182 USA; 3 Department of Genetics, Cell Biology and Anatomy, University of Nebraska Medical Center, Omaha, NE, 68198, USA

## Abstract

**Background:**

Nonhuman primates (NHPs) are essential for biomedical research due to their similarities to humans. The utility of NHPs will be greatly increased by the application of genomics-based approaches such as gene expression profiling. Sequence information from the 3' end of genes is the key resource needed to create oligonucleotide expression arrays.

**Results:**

We have developed the algorithms and procedures necessary to quickly acquire sequence information from the 3' end of nonhuman primate orthologs of human genes. To accomplish this, we identified terminal exons of over 15,000 human genes by aligning mRNA sequences with genomic sequence. We found the mean length of complete last exons to be approximately 1,400 bp, significantly longer than previous estimates. We designed primers to amplify genomic DNA, which included at least 300 bp of the terminal exon. We cloned and sequenced the PCR products representing over 5,500 Macaca mulatta (rhesus monkey) orthologs of human genes. This sequence information has been used to select probes for rhesus gene expression profiling. We have also tested 10 sets of primers with genomic DNA from Macaca fascicularis (Cynomolgus monkey), Papio hamadryas (Baboon), and Chlorocebus aethiops (African green monkey, vervet). The results indicate that the primers developed for this study will be useful for acquiring sequence from the 3' end of genes for other nonhuman primate species.

**Conclusion:**

This study demonstrates that human genomic DNA sequence can be leveraged to obtain sequence from the 3' end of NHP orthologs and that this sequence can then be used to generate NHP oligonucleotide microarrays. Affymetrix and Agilent used sequences obtained with this approach in the design of their rhesus macaque oligonucleotide microarrays.

## Background

Gene expression profiling is expected to rapidly increase the information yield from experiments using nonhuman primates (NHPs). This is important because NHPs are required for the study of AIDS, stem cell biology, reproduction and neuroscience, but are expensive and in short supply [1-14].

One question which must be addressed is: given the close evolutionary relationship between rhesus macaque and humans, why not use available human oligonucleotide microarrays with rhesus macaque samples? Human oligonucleotide microarrays have been used with chimpanzee samples to obtain useful information [15-17]. Cross-species hybridization experiments utilizing rhesus samples with human oligonucleotide microarrays have also been attempted [15,18,19]. Although useful information has been obtained, there are serious limitations to this approach. Cross-species comparisons introduce mismatches between a probe and a transcript that are not related to gene expression. Thus, it is impossible to know if a weak or absent signal is due to low levels of expression or to a mismatch. Approximately 40% of rhesus genes are not detected with a human GeneChip [18,19]. For genes that are scored present, the abundance of some transcripts may be underestimated due to mismatches between some of the human probes and rhesus targets. Longer probes might be expected to be more forgiving of mismatches, but even cDNA microarrays have a significant false negative rate when human microarrays are used with rhesus samples [20]. Thus, the use of human micorarrays with rhesus macaque samples results in a high rate of false negatives and does not allow for the acquisition of quantitative information. Clearly, a rhesus macaque specific expression array is needed.

Construction of oligonucleotide-based microarrays requires sequence from the 3' end of a transcript. There are two reasons for this. First, most sample labeling protocols are 3' biased [21,22]. As a result, probes chosen from sequence more than 1 kb from the 3' end of a gene may not detect a transcript. Second, it is important to choose probes from the 3' untranslated region (UTR) because such probes are most likely to be able to distinguish between gene family members. This is because coding sequences are much more highly conserved than 3' UTR [23].

We report a fast and efficient approach to obtaining high quality sequence of the 3' end NHP orthologs of human genes. The terminal exons of 15,401 well-annotated human genes were identified. Primer3 [24] was used to design primers that amplified at least 300 bp of 3' sequence. PCR was performed with these primers using rhesus macaque genomic DNA as the template. PCR products were cloned and sequenced. Over 5,300 rhesus macaque gene sequences have been deposited in GenBank. These sequences were used in the creation of rhesus macaque oligonucleotide microrarrays by two major companies, Affymetrix and Agilent. In addition, ten of the primer pairs designed in the course of this project were used with DNA obtained from three additional NHPs: cynomolgus monkey (*Macaca fascicularis*), baboon (*Papio hamadryas*), and African green monkey (*Cercopithecus aethiops*).

## Results

**Figure 1:**
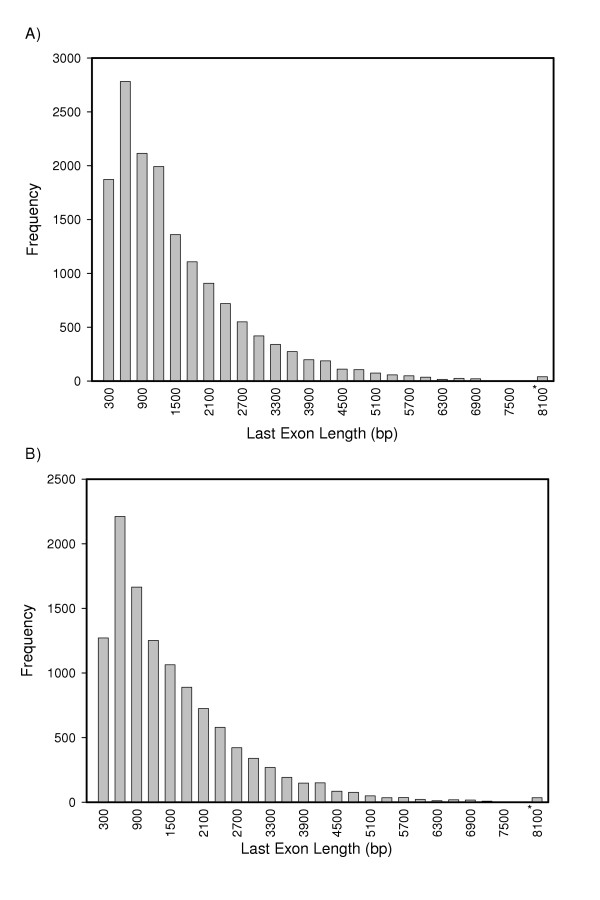
**Last exon lengths**. A) Lengths of human last exons (including incomplete exons). N = 15,401; mean = 1397.6; median = 1003; standard deviation = 1257.3. The values on the abscissa are the upper lengths of the bins; e.g., the bar at 600 bp includes last exons with lengths > 300 bp and ≤ 600 bp. The last bin – 8100 and marked with an asterisk (*) – contains last exons > 7800 bp in length. B) Lengths of human last exons (complete exons only). N = 11,502; mean = 1414.9; median = 1048; standard deviation = 1255.3. Only last exons obtained from mRNA that was complete on the 3' end are included (see discussion in the text for an explanation of how this was determined). The last bin – 8100 and marked with an asterisk (*) – contains all last exons with lengths > 7800 bp.

We found that at least 88% of human last exons were greater than 300 bp in length (Fig. 1A). The mean length for all last exons, including those that may be incomplete on the 3' end was 1,398 bp (N = 15,401). The median length was 1,003 bp. The shortest last exon in this group was 24 bp; the longest was 18,174 bp. Because the 3' end has not been determined for all transcripts, we also examined the lengths of complete last exons (N = 11,584; Fig. 1B). For these genes, the mean and median lengths were 1,414 bp and 1,046 bp, respectively. The shortest and longest last exons in this group were 27 bp and 18,174 bp, respectively. The last exon sequences we determined are available as Additional files 1, 2, 3.

PCR was used to amplify rhesus macaque genomic DNA using human primers. The PCR success rate (defined as yielding a correct-sized band with, at most, a few minor bands) with the first set of primers designed was 74%. If the first set of primers failed to amplify, a second pair of PCR primers was designed. The PCR success rate with the second set of primers was 59%. Thus, with no more than 2 sets of human primers, 90% of the rhesus macaque genes could be amplified. Only 2% of the PCR products proved difficult to clone. We have deposited sequence information for over 5,300 rhesus genes in the Sequence Tagged Site database (dbSTS) at NCBI. These sequences were used in the design of rhesus macaque oligonucleotide microarrays by Affymetrix and Agilent.

We chose ten primer sets that had worked successfully with rhesus macaque DNA to determine whether the same primer sets could be used with the genomic DNA of other NHPs. These ten genes were chosen to represent a range of identities between rhesus and human sequence – 91.21 to 98.57% identity (Table 1). The mean similarity between these 10 rhesus and human sequences was 94.42%. All PCRs performed with the 10 primers sets worked with both cynomolgus monkey and baboon DNA. Nine of the ten primer sets worked with vervet DNA. New primers were designed for the one gene (IFNG) that did not work with vervet DNA. The redesigned primers were successful with vervet DNA. The mean similarity to human sequence was about 94% for rhesus, cynomolgus monkey, baboon and vervet sequences. We also compared cynomolgus monkey, baboon and vervet sequences with rhesus sequences. Cynomolgus sequences were highly similar to rhesus sequences: mean 99.76% identical, range 99.48 to 100% identical. Most baboon sequences were also highly similar to rhesus sequences: mean 99.07% identical, range 97.85 to 99.64% identical. The vervet sequences were the least similar to rhesus sequences: mean 98.60% identical, range 97.26 to 99.74% identical.

**Table 1:** Percent identity of 10 sequences obtained using the primers developed for this project with genomic DNA from rhesus macaques, cynomologus monkeys, baboons and vervets.

	% identity with rhesus			% identity with human			
**Gene**	**Cyno**	**Baboon**	**Vervet**	**Rhesus**	**Cyno**	**Baboon**	**Vervet**
IGF1	100	99.61	99.74	98.57	98.57	98.12	98.57
ESR1	99.75	99.63	99.63	98.01	98.14	98.38	98.01
IFNG	99.48	99.13	98.62	96.52	96	96.52	96.17
DGKI	99.63	99.63	99.26	95.36	95.14	95.38	94.87
RNF2	99.72	99.02	99.02	94.44	94.16	94.44	94.85
ADRBK2	100	99.64	97.57	93.55	93.55	93.43	92.47
IL15RA	99.63	98.14	96.66	92.36	92.36	92.12	90.15
TNF	100	98.94	98.82	92.17	92.17	92.72	92.72
IL16	99.42	99.13	99.42	92.03	91.46	91.75	92.03
TYK2	100	97.85	97.26	91.21	91.21	91.41	91.6
							
Mean	99.76	99.07	98.6	94.42	94.28	94.43	94.14

## Discussion

### Last exons

Our use of genomic DNA as a template for targeted PCR is critically dependent on last exon length. At least 300 bp of sequence is preferred for the design of oligonucleotide probes present in oligonucleotide microarrays available from Affymetrix [25]. Because at least 88% of all human genes have last exons greater than 300 bp, for most genes, genomic DNA can be used as the PCR template for obtaining 3' sequence.

Our calculated mean and median lengths for all human last exons, 1,398 and 1,003, respectively are considerably longer than previous reports of last exon length [26-28], the longest mean of which was 811 bp. The procedures used to determine the lengths of last exons in these studies are not clear though most likely were based on aligning mRNA sequence with genomic sequence as done here. One possible reason for the longer last exons observed in the current study is that the sequence data necessary to determine the true 3'-end of many transcripts has become available only recently. Our results are based on the use of RefSeq Release 11 and GenBank Build 147, both obtained from NCBI on 23 May 2005; other reports are based on earlier versions of the genome when less 3' information was available. Although many of the transcripts in this release of RefSeq are annotated as being complete on the 3' end, we increased the number of complete transcripts by extending them as far as possible in the 3' direction by using additional mRNA sequences. An increase in the number of complete transcripts would obviously result in an increase in the mean last exon length. This is supported by the fact that when we considered only genes for which a 3' end was present, the mean and median values increased.

### Use of STS primers for other NHP species

Our results suggest that the primers developed for this project will be useful for obtaining sequence information from the 3' end of genes of other NHP species. Further, given that the rhesus and cynomolgus sequences were very similar, we predict that oligonucleotide microarrays designed based on rhesus sequence will work very well with cynomolgus samples. Baboon samples should also work well with rhesus oligonucleotide microarrays for most genes. Vervet sequences for some genes are more divergent from rhesus than cynomolgus monkey or baboon, as would be expected based on evolutionary relationships. Thus, the false negative detection rate observed with vervet samples on a rhesus oligonucleotide microarray may be significant and a targeted approach to obtaining 3' gene sequence from the vervet using primers developed for this project may be justified.

## Conclusion

This study demonstrates that human genomic DNA sequence can be leveraged to obtain sequence from the 3' end of NHP orthologs and that this sequence can then be used to generate NHP oligonucleotide microarrays. Affymetrix and Agilent used sequences obtained with this approach in the design of their rhesus macaque oligonucleotide microarrays.

## Methods

**Figure 2:**
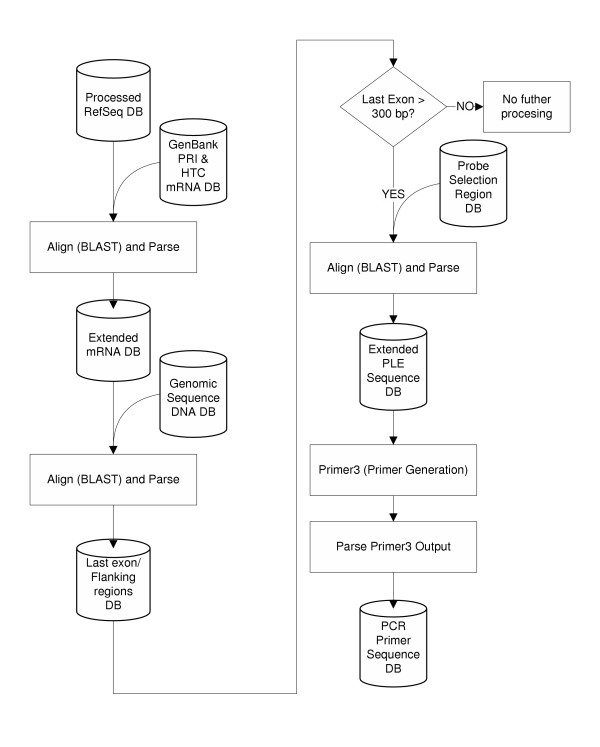
Flowchart demonstrating the process used to obtain primer pairs for the amplification of NHP orthologs of human genes.

Figure 2 illustrates the project flow for generating PCR primers. A more complete overview is presented in Additional file 4.

### Determination of last exons

Human mRNA reference sequences (RefSeq; Release 11) were obtained from NCBI [29]. Sequences corresponding to non-protein coding, withdrawn, hypothetical or pseudogenes in the LocusLink template database  [30] were removed. To create a non-redundant dataset of mRNAs, only the longest RefSeq per gene was retained. After these operations, our dataset contained a total of 15,663 unique transcripts; accession numbers for these sequences are available in Additional file 5.

Because 3' sequence is used to select probes, we aligned (BLAST) RefSeqs with all human mRNA sequences from the GenBank Primate (PRI) and high-throughput cDNA sequencing (HTC) division to extend the 3' end of the transcripts as far as possible. A given RefSeq was extended with the mRNA sequence that: 1. had at least 300 bp of alignment with the 3' end of the RefSeq; 2. had at least 98% identity with the RefSeq in the region of alignment; and 3. extended the RefSeq the furthest in the 3' direction.

The extended mRNA sequences obtained above were aligned with genomic DNA sequences from the phase 2 and 3 Human Genome Project sequence databases  [31]. The approximate boundaries of exons can be determined by examining High Scoring Pairs (HSP) from the alignment. Because HSPs only deviate from true exons by a few nucleotides (due to ambiguity in the alignment at the splice site), we defined the last exon as the 3'-most HSP. Both last exons and flanking sequences were recorded. We were unable to determine the last exon for 367 genes.

### Determination of completeness

The 3' end has not been determined for all transcripts. To calculate accurate statistics regarding last exon length, it is important to work with a dataset that contains complete last exons; this requires knowledge of which transcripts are complete on the 3' end. To determine whether transcripts were complete on the 3' end, we used two strategies. First, we examined the complete GenBank FlatFile records of the RefSeqs. Transcripts were considered complete on the 3' end if: 1. the Comment field contained the phrase: "COMPLETENESS: full length" or "COMPLETENESS: complete on the 3' end"; or 2. the Feature table contained the keys "polyA_signal" or "polyA_site". Second, because not all complete transcripts have been annotated as such in GenBank, we also aligned the 3' end of the extended mRNA sequence with genomic sequence (hs_phase3). If three or more "A"s were found on the 3' end of the transcript that were not present in the corresponding genomic sequence, we assumed that this indicated a poly-A tail was present in the extended mRNA sequence and that therefore the transcript was complete on the 3' end. We defined full length last exons as those derived from mRNA sequences that were complete on the 3' end.

### Primer selection procedure

**Figure 3:**
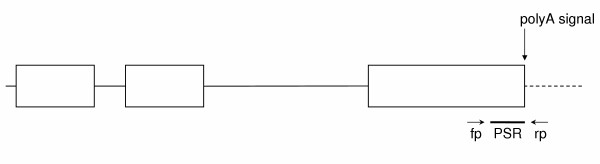
**Diagram illustrating the strategy for designing primers to amplify 3' sequence from NHP genes**. Exons are indicated by boxes. Solid lines represent introns. The dashed line indicates sequence 3' to the 3' end of the gene. The poly-A signal is indicated at the 3' end of the last exon. fp = forward primer; rp = reverse primer; PSR = probe selection region.

Affymetrix has identified Probe Selection Regions in the 3' ends of transcripts which are frequently contained within the last exon of genes. We aligned (BLAST) the last exons with the Probe Selection Regions. If there was at least 300 bp of alignment between the Probe Selection Region and the last exon, Primer3 [24] was used to select primers that flanked the Probe Selection Region (Figure 3). If not, Primer3 was used to amplify at least 300 bp of sequence from the last exon. The Human Mispriming library was selected; when this option is chosen, Primer3 screens out interspersed repeats from sequences which can be used for primers.

### Bioinformatics

Automation of the Determination of Last Exons and Primer Selection procedures was accomplished with software written in Python and Java and employed packages from the  site. An archived collection of Java methods used in determining last exons and generating primer pairs is included as Additional file 6. A complete description of the procedures used to determine last exons and design primers can be found in Additional file 7. A detailed description of the quality control procedures used to verify the results obtained for last exon determination and primer design can be found in Additional file 8. Data was stored and organized in PostgreSQL and Filemaker Pro databases. All primer sequences, PCR conditions and sequences generated as a result of this project have been deposited in GenBank (see Additional file 9 for accession numbers).

### PCR

Genomic DNA was isolated from the liver of a one year old male rhesus macaque at the Oregon National Primate Research Center. Primers were synthesized by Sigma-Genosys (The Woodlands, TX) or IDT (Coralville, IA). Primers were resuspended in RNAse/DNAase free water to 50 picomoles/μl. Primers were then aliquoted into 96-well daughter plates with a Biomek 2000 robot (Beckman-Coulter). 10 μl of RNAse/DNAse free water, 1 μl of forward and reverse primers at 50 picomoles/μl and 2 μl of genomic DNA at 100 ng/μl were dispensed by the robot into each well of a 96-well PCR plate (MJ Research). A mastermix was prepared that included PCR buffer, dNTPs and water and was dispensed into each PCR well such that the final concentration was 1× PCR buffer and 200 μM dNTPs. 0.5 μl (2.5 units) of Fast Start High Fidelity Polymerase (Roche) was then added to each well. The PCR plate was placed in a MJ Research PTC-100 thermocycler and the following program used: Step 1. 95°C for 2 minutes; Step 2. 95°C for 30 sec, 51°C for 30 sec, 72°C for 1 min, 35 cycles; Step 3. 72°C for 7 minutes. For the primers that failed the first PCR, PCR conditions were altered. If the first PCR resulted in no band, the annealing temperature was decreased to 48°C. If the first PCR resulted in multiple bands, the annealing temperature was increased to 53°C. All PCR cleanups were done using the QIAquick 96-Multiwell PCR Purification System (Qiagen).

### Cloning and DNA purification

Most PCR products were cloned into pGEM-T Easy (Promega). Some PCR products were cloned into pCR-TOPO XL (Invitrogen). After transformation, cells were incubated in SOC medium for 2 hours at 37°C at 180 RPM. 50 μl of the cell suspension was added to 35 mm LB-agar plates and incubated overnight at 37°C. Clones were picked and grown in 2xTY growth media and incubated overnight at 37°C at 300 RPM. Plasmid DNA was purified using the QIAprep 96 Turbo Miniprep Kit (Qiagen).

### Sequencing and Genbank deposits

All clones were sequenced in both directions on an ABI3130 Genetic Analyzer using m13 forward and reverse primers. Sequences were aligned, edited in Sequencher (Gene Codes Corporation, Ann Arbor, MI) and BLASTed to check identity and percent homology with the targeted human homolog. The human primers were deleted from the edited sequence and the edited sequence deposited in GenBank following the standard STS format. STS files were generated from a Filemaker Pro database using the Troi File Plug-in 32. A list of accession numbers is provided as Additional file 9.

## Authors' contributions

RBN proposed the project, developed the algorithms and wrote the manuscript. MAP designed and implemented the software used in primer selection and determination of last exon, participated in the analysis of the exon lengths and contributed to the writing of the manuscript. ERS assisted with development of the project, designed the strategies for and supervised the sequencing, sequence analysis, annotation and deposition and contributed to the writing of the manuscript. YJ and CG assisted with sequencing, sequence analysis and annotation. ST and NB assisted with the PCR, cloning, DNA preparation and data organization. SRO assisted with the development of the project.

## Note

Website Reference

; Rhesus GeneChip Information

## Supplementary Material

Additional File 1 Last exon sequences (Part 1). FASTA-formatted files containing the last exons of 15,401 unique human genes. XML-styles tags in the header are used to denote the LocusLink ID (now GeneID) and symbol of the corresponding gene. The tags <baseAccNo> and <extAccNo> denote the accession number of the base reference sequence and mRNA sequence, respectively, used to generate the extended mRNA sequence from which the last exon was derived. Click here for file

Additional File 2 Last exon sequences (Parts 2). FASTA-formatted files containing the last exons of 15,401 unique human genes. XML-styles tags in the header are used to denote the LocusLink ID (now GeneID) and symbol of the corresponding gene. The tags <baseAccNo> and <extAccNo> denote the accession number of the base reference sequence and mRNA sequence, respectively, used to generate the extended mRNA sequence from which the last exon was derived. Click here for file

Additional File 3 Last exon sequences (Parts 3). FASTA-formatted files containing the last exons of 15,401 unique human genes. XML-styles tags in the header are used to denote the LocusLink ID (now GeneID) and symbol of the corresponding gene. The tags <baseAccNo> and <extAccNo> denote the accession number of the base reference sequence and mRNA sequence, respectively, used to generate the extended mRNA sequence from which the last exon was derived. Click here for file

Additional File 4 Procedure Flowchart. Provides a detailed overview of the procedure used to obtain primer pairs for the amplification of NHP orthologs of human genes. Click here for file

Additional File 5 Accession numbers of Unique Reference Sequences (with LocusLink ID, now GeneID, and Gene Symbol). List of accession numbers of the 15,633 unique human mRNA Reference Sequences used to generate the human last exons in this study; includes the associated LocusLink ID (2^nd ^column) and gene symbol (3^rd ^column) for each sequence. Click here for file

Additional File 6 Java methods. An archived collection of Java methods used in determining last exons and generating primer pairs. Used in conjuction with bioJava 1.30 (also included). See the file BlastObjectParser.java for a brief explanation on usage of the various processing methods. Click here for file

Additional File 7 Detailed Methods. Contains the complete procedures used to determine last exons and generate primer pairs. Details left out for brevity in the main text are provided here. Click here for file

Additional File 8 Quality control. Provides a detailed description of the quality control measures employed when generating last exons and primer pairs. Click here for file

Additional File 9 List of STS Accession Numbers. List of GenBank accession numbers of the STS sequences generated in this study. Click here for file
