# Supplementary figures and images for: Leveraging human genomic information to identify nonhuman primate sequences for expression array development
Source: BMC Genomics. 2005 Nov 15;6:160. doi: 10.1186/1471-2164-6-160 (PMC1314899; doi:10.1186/1471-2164-6-160)

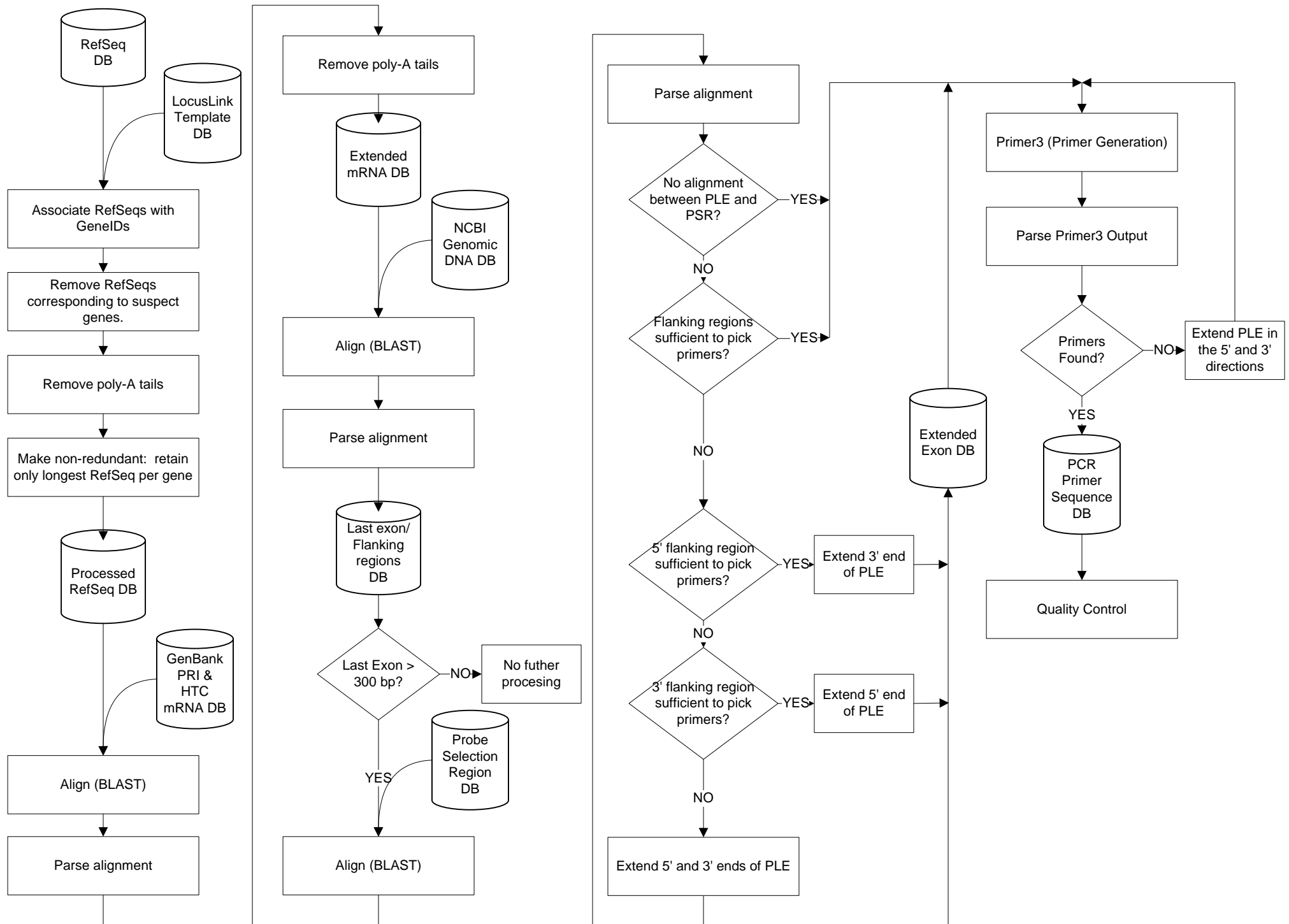

Supplement: Additional File 4 — Procedure Flowchart. Provides a detailed overview of the procedure used to obtain primer pairs for the amplification of NHP orthologs of human genes. [file 1471-2164-6-160-S4.pdf]
