# Supplementary material for: Leveraging human genomic information to identify nonhuman primate sequences for expression array development
Source: BMC Genomics. 2005 Nov 15;6:160. doi: 10.1186/1471-2164-6-160 (PMC1314899; doi:10.1186/1471-2164-6-160)
Supplement: Additional File 7 — Detailed Methods. Contains the complete procedures used to determine last exons and generate primer pairs. Details left out for brevity in the main text are provided here. [file 1471-2164-6-160-S7.pdf]

Detailed Methods

Following are the complete procedures we used to determine last exons and generate primer pairs. Details left out in the main text for brevity are provided here.

<u>Determination of Last Exons</u>

In brief, the last exon of human genes was obtained by aligning extended reference sequences (RefSeqs) with genomic DNA.

Step 1: Process mRNA sequences. Human mRNA RefSeqs were obtained from NCBI (*ftp://ftp.ncbi.nih.gov/refseq/H_sapiens/mRNA_Prot/human.rna.fna.gz*). RefSeqs were associated with GeneIDs (formerly LocusLink IDs) and official symbols using the LocusLink template database (LL_tmpl; *ftp://ftp.ncbi.nih.gov/refseq/LocusLink/LL_tmpl.gz*); note that as of 1 June 2005, this database is no longer being updated. RefSeqs corresponding to "suspect" genes—pseudogenes, withdrawn and hypothetical genes, and genes that were not protein-coding—were filtered out. For some of the RefSeqs, poly-A tails were recorded. To avoid errors in alignment, if there were three or more "A"s at the 3' end of a RefSeq, all but two were removed; single, non-"A"s in the midst of a putative poly-A tail were ignored. In cases where multiple RefSeqs were associated with the same GeneID, only the longest RefSeq was retained.

Step 2: Create extended mRNA sequences. The RefSeqs in 1) were aligned (BLAST version 2.2.9) with a cumulative RNA database (query and target, respectively) formed using human mRNA records from the GenBank Primate (PRI) and high-throughput cDNA (HTC) divisions. For a given alignment, the mRNA that had at least 300 bp of alignment on the 3' end of the RefSeq with at least 98% identity and that extended the farthest in the 3' direction was used to extend the reference sequence. This was done by adding the 3' end of the mRNA—everything

downstream from the alignment with the reference sequence—on to the end of the RefSeq. This created an extended mRNA sequence.

Step 3: Extract Last Exon and Flanking Sequences. After removing the poly-A tails from the sequences in 2), BLAST (version 2.2.9) was used to align the extended mRNA sequences (query) with human genomic DNA sequences (target) from the human phase 2 and 3 Genome Project sequence databases (*ftp://ftp.ncbi.nih.gov/genbank/genomes/H_sapiens/hs_phase[23].fna.gz*). The output of the alignment was parsed as follows: For a given extended mRNA sequence, the first high-scoring pair (HSP) in which the target sequence extended to within five nucleotides of the end of the mRNA sequence and in which the percent identity was 98% or greater was taken as the last exon. For genes in which a last exon was not found the distance was increased from five to twenty-five bp and the output of the alignment was reparsed. Last exons with lengths of fifty bp or less were considered "suspect;" these were examined manually and corrected as necessary (in most cases, the incomplete removal of a putative poly-A tail—e.g., due to sequential non-"A" characters—resulted in an incorrect HSP being chosen as the last exon by our software). 100 bp of sequence 5' to the last exons and 200 bp of sequence 3' to the last exons were recorded. Since Affymetrix requires at least 300 bp of sequence to choose probes, any last exon with length less than 300 bp was not processed further.

Primer Selection

The program Primer3 was used to design primers that amplified DNA that included the 3' end of the terminal exon of the target genes.

Step 1: Determine Target Sequences. For each gene Affymetrix has identified Probe Selection Regions from which to pick probes. These sequences are 3'-biased and are frequently contained

within the last exon. We aligned (BLAST) the last exons obtained using the above procedure (query) with human Probe Select Region sequences obtained from Affymetrix (target). The range of the Probe Selection Region within the last exon was recorded. If there was at least 100 bp of sequence 5' and 3' flanking the probe selection region, the last exon itself was considered the target sequence. If there was not at least 100 bp of sequence within the last exon 3' to the probe selection region, 200 bp of genomic sequence was added to the 3' end of the last exon to generate a 3'-extended target. If there was not at least 100 bp of sequence within the last exon 5' to the probe selection region, 100 bp of genomic sequence was added to the 5' end of the last exon to generate a 5'-extended target. In a few cases, it was necessary to extend the exon in both the 5' and 3' directions.

Step 2. Primer Generation. Primer3 was used to select primers that flanked the probe selection region. Range information from Step 1) was used to determine the region to be amplified. If there was less than 300 bp of alignment between the probe selection region and the last exon, no attempt was made to amplify the probe selection region; instead, Primer3 parameters were chosen to amplify at least 300 bp of sequence from the last exon. In all cases, the human Mispriming Library was used.
