# Supplementary material for: Leveraging human genomic information to identify nonhuman primate sequences for expression array development
Source: BMC Genomics. 2005 Nov 15;6:160. doi: 10.1186/1471-2164-6-160 (PMC1314899; doi:10.1186/1471-2164-6-160)
Supplement: Additional File 8 — Quality control. Provides a detailed description of the quality control measures employed when generating last exons and primer pairs. [file 1471-2164-6-160-S8.pdf]

Quality Control

Several quality control measures were implemented to insure that the primer pairs generated using our procedure were correct; they are described here.

<u>Determination of Last Exons</u>

The last exon (query) was aligned (BLAST; 2.2.9) against the reference sequence database (target). For a given alignment, the accession number of one of the high scoring pairs (HSP) with 100% identity was required to match the accession number of the query; the sense of the alignment was required to be plus/plus.

<u>Primer Selection</u>

The last exon used to generate a given primer pair (query) was aligned (BLAST) against genomic DNA (target). The first HSP of the first hit was required to have a percent identity of at least 98% and the identity (accession number) of the target sequence of this hit was required to match that as having been used to extend the last exon. In addition, the alignment had to be uninterrupted.

The generated primers (query) were aligned (BLAST) against the reference sequence database (target). For a given primer, if a hit whose recorded gene identity matched that recorded for the primer was found, the sense and position of the alignment were examined and checked for correctness. Specifically, for primers for genes with no 5' or 3' extension to the last exon, both primers were required to exist in the reference sequence and be in the correct sense; i.e., plus/plus for the forward primer and plus/minus for the reverse. For primers for genes with only a 3' extension to the last exon (no 5' extension), the forward primer was required to exist in the reference sequence and be in the correct sense (plus/plus); if the reverse primer existed in the

reference sequence, it was required to be in the correct sense (plus/minus). Finally, for genes with last exons having both a 5' and 3' addition, neither primer was required to exist in the reference sequence; if they did, they were required to be in the correct sense.

For later reference, the following information was recorded for all primer pairs: 1) The position of the probe selection region (if used) in the last exon; 2) The position of the last exon in the genomic clone; 3) The position of the last exon in the target sequence; 4) The position of the target sequence in the genomic clone; 5) The position of the probe selection region in the target sequence; 6) The position of the target sequence in mRNA; 7) The position of the forward and reverse primers in the target sequence; and 8) The position of the forward and reverse primers in the reference sequence (may have been null if the primer existed in an extended region).
